# UCP-2 is involved in angiotensin-II-induced abdominal aortic aneurysm in apolipoprotein E-knockout mice

**DOI:** 10.1371/journal.pone.0179743

**Published:** 2017-07-06

**Authors:** Peng Yan, Ken Chen, Qiang Wang, Dachun Yang, De Li, Yongjian Yang

**Affiliations:** Department of Cardiology, Chengdu Military General Hospital, Chengdu, Sichuan, P.R. China; Maastricht University, NETHERLANDS

## Abstract

UCP-2 shows an important role in modulating of mitochondrial membrane potential and cell apoptosis. Whether or not UCP-2 could been a critical factor in preventing AAA formation is not known. We report that UCP-2 protein and mRNA expression were significantly higher in Ang-Ⅱ-induced AAA of mice. The incident rate of AAA in UCP-2^-/-^ApoE^-/-^ mice after Ang-Ⅱtreatment was higher than the rate in the UCP-2^+/+^ApoE^-/-^ mice. The abdominal aorta from UCP-2^-/-^ApoE^-/-^ mice showed the medial hypertrophy, fragmentation of elastic lamellas and depletion of α-SMA. The NADPH oxidase activity and level of MDA was significantly higher in UCP-2^-/-^ApoE^-/-^ mice than UCP-2^+/+^ApoE^-/-^ or WT mice. Besides, the SOD activity is increased in UCP-2^+/+^ApoE^-/-^ mice as compared with WT mice, whereas deficiency of UCP-2 decreased the increasing SOD activity in Ang-Ⅱ treated ApoE^-/-^ mice. UCP-2 knockout up-regulated the MMP2 and MMP9 expression in aortic aneurysm. Ang-Ⅱ induced apoptosis of VSMCs was increased in UCP-2^-/-^ApoE^-/-^ mice. And the expression of eNOS in vascular tissue from UCP-2^-/-^ApoE^-/-^ mice is lower than WT and UCP-2^+/+^ApoE^-/-^ mice. This study provides a mechanism by which UCP-2, via anti-oxidants and anti-apoptosis, participates in the preventing of AAA formation.

## Introduction

Abdominal aortic aneurysms (AAA), which occurs mostly among men older than 65 years of age, is the thirteenth leading cause of death in the USA [1]. The morbidity is estimated at between 1.3% to 8.9% in man and between 1.0% to 2.2% in woman [2]. The rupture of AAA leads to death in 65% of patients [2,3]. The aberrant interaction between genetic and environmental factors plays a key role in the pathological process of AAA [4]. The accumulation of reactive oxygen species (ROS) is one of the most important environmental factors leading to AAA. The increasing production of ROS in vascular wall induces inflammation and the elastic media degradation, and finally resulting in aortic rupture [5,6]. Previous study showed that the ROS up-regulates the proteolytic enzymes of extracellular matrix, and increases the matrix degradation and remodeling in human AAA biopsies [7].

While AAA may be caused not only by increased activity of ROS systems but also by defects in anti-ROS systems that serve as counter-regulatory mechanisms. The decreasing antioxidant enzymes including haem oxygenase (HO), superoxide dismutase (SOD), thioredoxin (TRX) and catalase results in excessive ROS [8,9]. Moreover, mitochondrial uncoupling proteins (UCPs) have been known as antioxidants to protect against oxidative damage via ROS homeostasis maintenance, which are the mitochondrial anion carriers and located in the mitochondrial membrane [10–12]. There are three UCP subtypes, including UCP-1, UCP-2 and UCP-3 [12,13]. Among those members of UCPs, UCP-2 is expressed widely in several tissues as skeletal muscle, heart and vascular cells, besides the white adipose tissue [14,15]. UCP-2 shows an important role in modulating of mitochondrial membrane potential (MMP). And previous study indicated that increasing expression of UCP-2 in vascular cells may prevent the development and progression of atherosclerosis in patients with increased ROS[16]. Besides, UCP-2 has been well-established as an apoptosis suppressor in different cell systems [17,18] while vascular smooth muscle cells apoptosis has been documented in the aortic aneurysms and dissections [19,20]. Thus, we supposed that UCP-2 could been a critical factor in preventing AAA formation via anti-oxidants and anti-apoptosis. To determine this hypothesis, a UCP-2 and apolipoprotein E (apoE) double-knockout mice was used to determine the effect of UCP-2 to the pathology of AAA.

## Methods

### The generation of UCP-2 and ApoE double-knockout mice and AAA model

The UCP-2^-/-^ApoE^-/-^ mice were generated by crossbreeding UCP-2 null mice with ApoE null mice (Jackson Laboratory, Bar Harbor, ME, US) as previously described [21]. The genotyping in the knockout mice was verified by PCR. And DNA samples were obtained from toes or tails of the mice. The forward primer of UCP-2 mutant was GCTCTGAGCCCTTGGTGTAG and the reverse primer was GCTCTGAGCCCTTGGTGTAG (Jackson Laboratory Protocol, stock NO. 005934). The forward primer of ApoE mutant was GCCTAGCCGAGGGAGAGCCG and the reverse was GCCGCCCCGACTGCATCT (Jackson Laboratory Protocol, stock NO. 002052).

As described previously[22], the AAA of UCP-2^+/+^ApoE^-/-^ or UCP-2^-/-^ApoE^-/-^ mice was induced by chronic infusion of 1000 ng/kg/min angiotensin Ⅱ (Ang-Ⅱ, sigma, St. Louis, USA) via mini-osmotic pumps (Model 2004, Durect, Cupertino, CA) in 8-week-older mice for 4 weeks. Mice were anesthetized with sodium pentobarbital (30 mg/kg) for implantation of mini-osmotic pumps. Blood pressure was measured weekly using the Visitech tail cuff system (Apex, NC, USA). Then, the mice were euthanized by cervical dislocation. The aneurysmal portion of the aorta was removed for following experiment.

This study was approved by the Research Council and Animal Care and Use Committee of Chengdu Military General Hospital. All experiments were conformed to the guidelines of the American Association for the Accreditation of Laboratory Animal Care and conformed to the guidelines of the ethical use of animals, and all efforts were made to minimize animal suffering and to reduce the number of animals used.

### Immunoblotting

Aortic tissue, cleared of blood with phosphate buffer saline (PBS), and lysed in lysis buffer. After centrifugation, the tissue homogenates were collected and separated by SDS-polyacrylamide gel and transferred to polyvinylidene fluoride (PVDF) membranes. After washing with Tris-buffered saline Tween-20 (TBST) and blocking with 5% milk powder, the transblots were probed with the rabbit anti-UCP-2 antibody (1:500, abcam, Cambridge, MA), rabbit anti-MMP2 (1:500, abcam, Cambridge, MA) and MMP9 antibody (1:500, abcam, Cambridge, MA) at 4°C overnight. The membranes were then washed and detected with goat anti-rabbit-IgG (1:4000, BOSTER, Wuhan, China) conjugated to horseradish peroxidase, and the bands were visualized with enhanced chemiluminescence (Millipore, Billerica, MA). The amount of protein transferred onto the membranes was verified by immunoblotting for GAPDH (rabbit anti-GAPDH antibody, 1:1000, abcam, Cambridge, MA).

### RNA extraction and PCR

The total RNA from abdominal aortic tissue were extracted by Trizol (Life Technologiese Invitrogen, Shanghai, China). A total 1 μg of RNA was used to synthesize cDNA and served as a template for amplification of UCP-2 by RNA reverse transcriptase Kit (Takara, Dalian, China). The forward primer of UCP-2 was 5’-AACAGTCCCAGACAGCCTACA-3’ and the reverse primer was 5’-CCTTCTTTCACTCCCATTTCC-3’ (767bp). The amplification was performed by Quantitative Real-time PCR by a SYBR green premix according to the manufacturer’s guide (SYBR Real-Time PCR Kit, Takara, Dalian, China). Mouse 18S ribosomal RNA were used as endogenous controls. Relative expressions of target genes were standardized to GAPDH, evaluated by the 2^-ΔΔCT^ method and given as a ratio to control the experiments.

### Histological analysis

After perfusion with saline containing 0.4% heparin, the aorta from mice were cleared of blood with PBS, kept in 4% paraformaldehyde, and then embedded in paraffin for sectioning and mounting on slide. After deparaffinizing and rehydrating by xylene and different concentration ethanol, the sections were stained with hematoxylin and eosin (H&E) under standard protocol, or treated with rabbit anti-α-SMA antibody (1:200, Santa Cruz) for immunohistochemistry. Moreover, the studies applied the Verhoeff-Van Gieson (VVG) stain for elastin measurement.

### Oxidative stress analysis

The ROS level was also evaluated with the dihydroethidium (DHE, Beyotime, China) staining. The frozen sections of aortic tissue were stained with DHE for 20 min and observed with a fluorescence microscope at excitation wave-lengths of 490 nm and emission of 590 nm. The fluorescence intensity of ROS was quantified by ImageJ (NIH, Bethesda, MD). Besides, the level of oxidative stress was determined via measurement of NADPH oxidase activity, malondialdehyde (MDA) concentration and SOD activity. The tissue homogenates were used to detect NADPH oxidase activity with diphenyleneiodonium (DPI), detect MDA with thiobarbituric acid (TBA) and detect SOD with nitroblue tetrazolium (NBT), following the manual of each assay kit (Beyotime, China).

### Statistical analysis

Comparison within more than two groups was made by repeated measures one-way ANOVA, and comparison among groups was made by factorial ANOVA with Holm-Sidak test. The data are expressed as mean ± SEM. And a value of *P<*0.05 was considered significant.

## Results

### UCP-2 was involved in the formation of AAA

**Fig 1:**
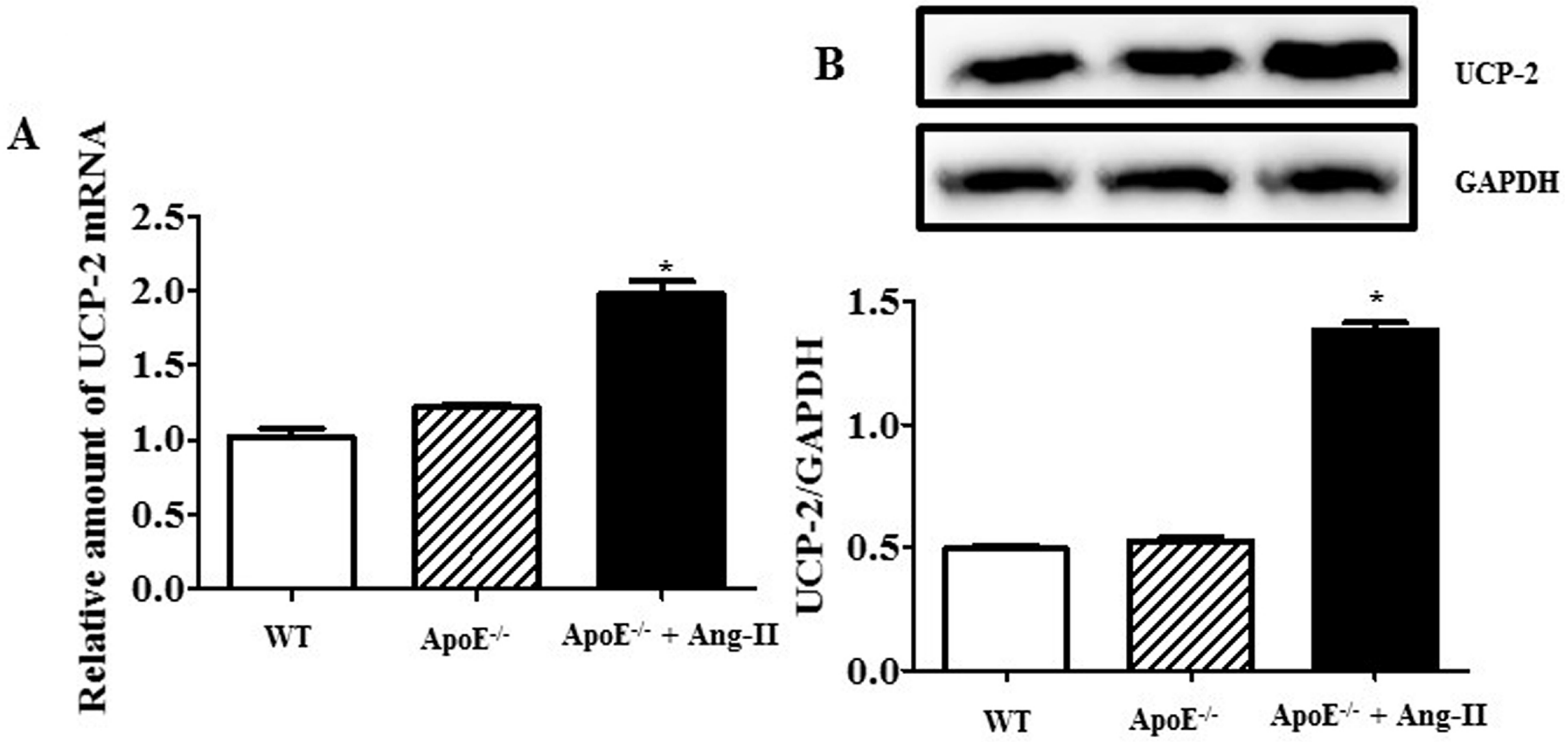
**UCP-2 mRNA (A) and protein (B) expression in AAA.** Results are expressed as the ratio of UCP-2 and GAPDH. (n = 5, *P<0.05 vs. others).

We first determined if UCP-2 expression showed any differences in the aortic aneurysm. The data found that the UCP-2 protein and mRNA expression were significantly higher in Ang-Ⅱ-induced AAA of mice (**Fig 1A**), indicating that the changes of UCP-2 expression in AAA occurred at both post-translational and transcriptional levels.

**Fig 2:**
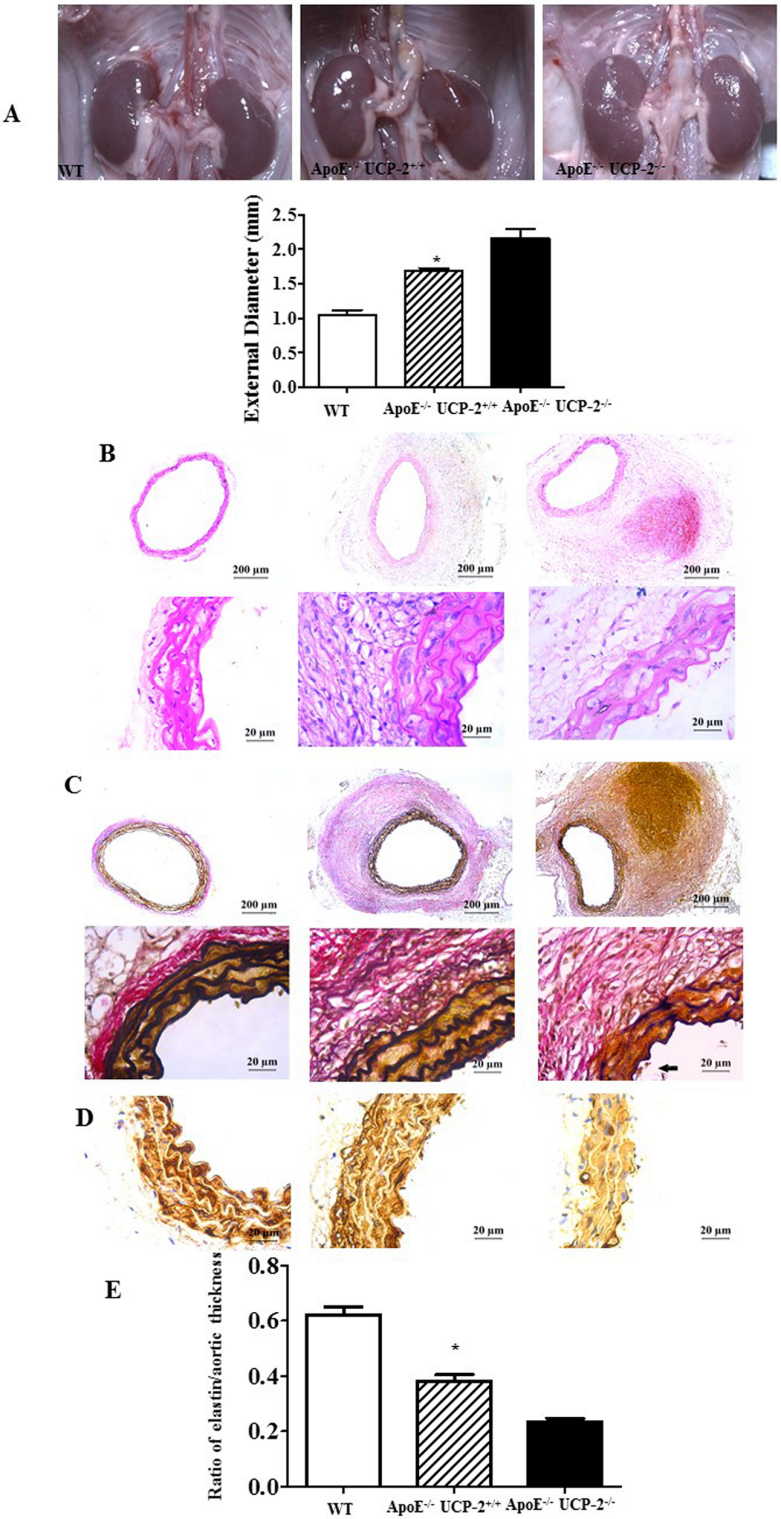
The deficiency of UCP-2 in AAA development. (**A**) Macroscopic views of the abdominal aorta. A representative photograph for each group to show the internal diameter of the abdominal aorta. The values represent the means ± SEM, n = 6 in each group. (*P<0.05 vs. others). (**B**) Hematoxylin and eosin staining of aorta. (**C**) Verhoeff-Van Gieson (VVG) staining of elastic fibers of the aneurysm sections. (**D**) Immunohistochemical staining of α-SMA to view the thickness of smooth muscle. (**E**) The ratio of elastin lamellae to aortic thickness. (n = 6, *P<0.05 vs. others).

Moreover, a UCP-2^-/-^ApoE^-/-^ mice model of AAA was generated. The systolic blood pressure of mice from each group increases in a time-dependent manner after Ang-II infusion (0 to 28 days). And systolic pressure in wt, ApoE knockout and ApoE/UCP-2 double-knockout group do not show any significant differences (**S1 Fig**). The incident rate of AAA in UCP-2^-/-^ApoE^-/-^ mice after Ang-Ⅱ treatment was 83.9% (26 of 31), higher than the rate in the UCP-2^+/+^ApoE^-/-^ mice (55%, 11 of 20). And the aortic expansion of UCP-2^-/-^ApoE^-/-^ mice was significantly increased as compared with the UCP-2^+/+^ApoE^-/-^ mice (**Fig 1B**). The histological studies showed the medial hypertrophy, fragmentation of elastic lamellas and depletion of α-SMA in abdominal aorta from UCP-2^-/-^ApoE^-/-^ mice (**Fig 2A–2C**). The ratio of elastin lamellae to aortic thickness is lower in aorta of UCP-2^-/-^ApoE^-/-^ mice than the UCP-2^+/+^ApoE^-/-^ or WT mice (**Fig 2D**). The data indicated that UCP-2 could play an important role in the formation of AAA.

### The deficiency of UCP-2 elevated oxidative stress of aorta

**Fig 3:**
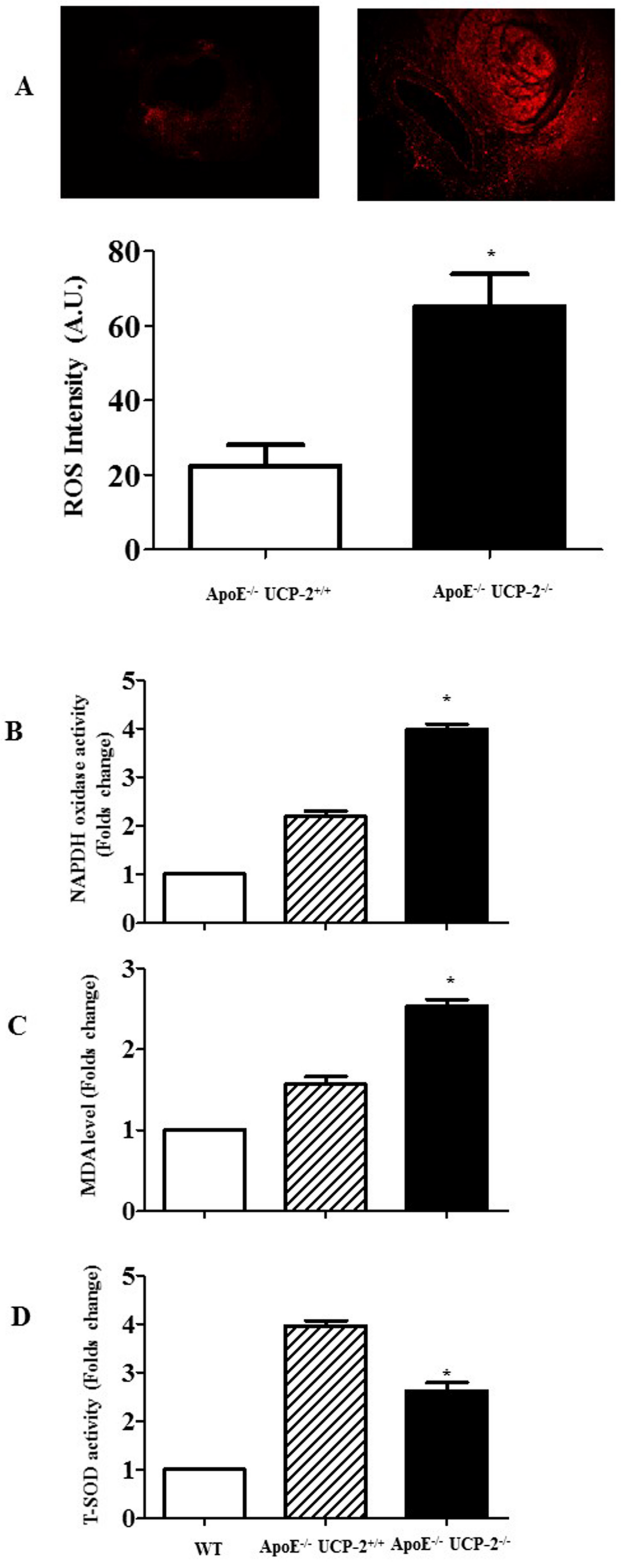
The deficiency of UCP-2 to ROS production in AAA tissue. (**A**) Effect of UCP-2- deficiency on ROS production in AAA. The ROS production was determined by DHE staining (red). The bar graph showed that ROS intensity of fluorescence activities in different groups (*P<0.05 *vs*. others, n = 5). (**B**) NADPH oxidase activity. (*P<0.05 *vs*. others, n = 5). (**C**) MDA level of AAA tissues. (*P<0.05 *vs*. others, n = 5) (**D**) Total SOD activity. (*P<0.05 *vs*. others, n = 5).

To verify the effect of ROS in AAA of UCP-2^-/-^ApoE^-/-^ mice, we evaluated the level of oxidative stress in aortic tissue. And the superoxide production, determined by fluorescent dye DHE staining, was increased in aorta from UCP-2^-/-^ApoE^-/-^ mice (**Fig 3A**). While the NADPH oxidase activity and level of MDA was significantly higher in UCP-2^-/-^ApoE^-/-^ mice than UCP-2^+/+^ApoE^-/-^ or WT mice (**Fig 3B** and **3C**). Besides, the SOD activity is increased in UCP-2^+/+^ApoE^-/-^ mice as compared with WT mice, whereas deficiency of UCP-2 decreased the increasing SOD activity in Ang-Ⅱ treated ApoE^-/-^ mice (**Fig 3D**), indicating that the antioxidant defense is impaired due to UCP-2 deficiency, and leading AAA formation. Collectively, our data found a protective effect of UCP-2 against Ang-Ⅱ induced oxidative stress and AAA.

**Fig 4:**
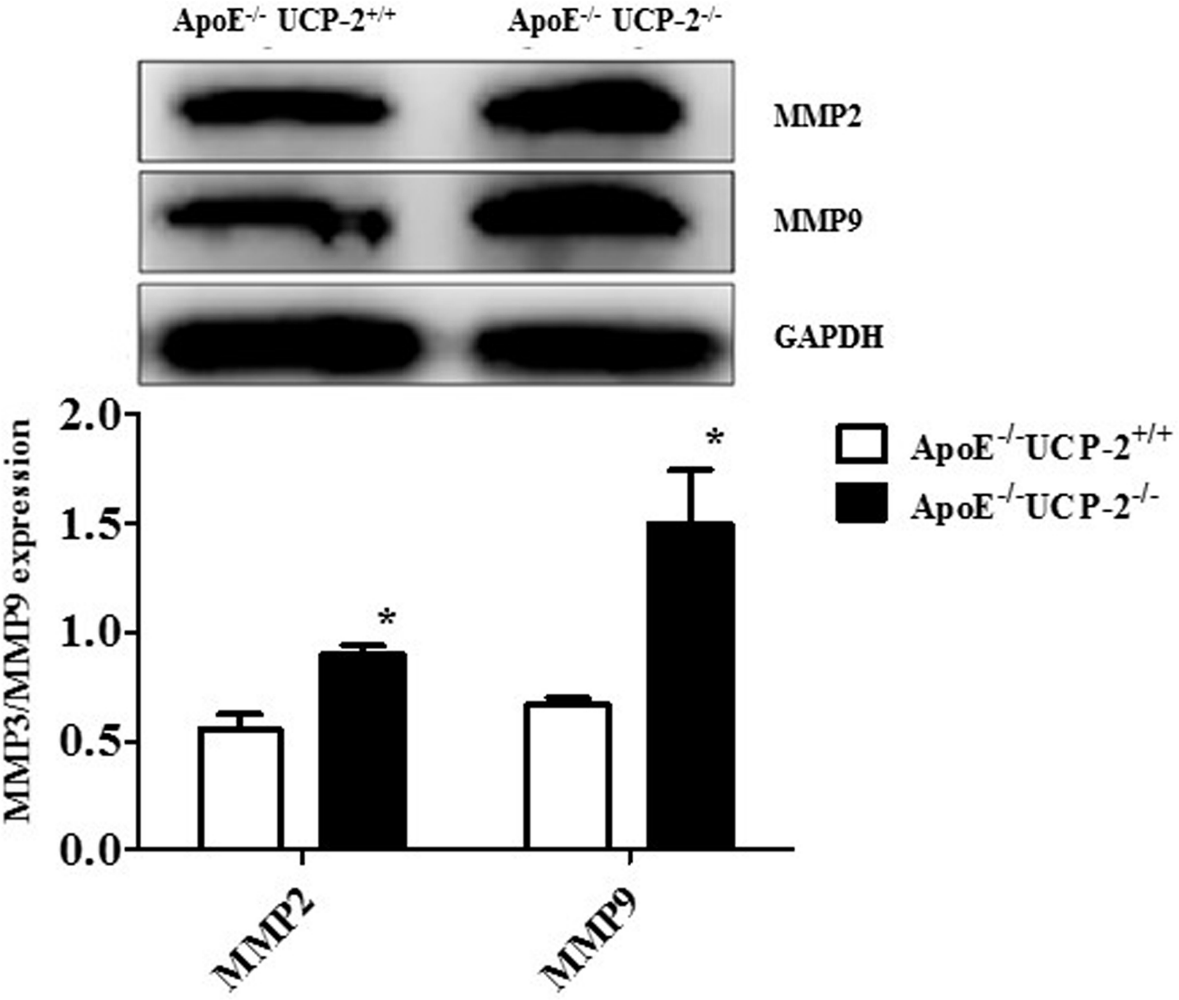
The effect of UCP-2- deficiency to MMP 2 and 9 protein expression in Ang-II-induced AAA mice. (*P<0.05 *vs*. others, n = 6).

Previous study showed that the ROS up-regulates the proteolytic enzymes of extracellular matrix and increases the matrix degradation [7]. Indeed, our results also showed UCP-2 knockout up-regulated the MMP2 and MMP9 expression in aortic aneurysm (**Fig 4**).

### AAA-associated cell apoptosis is aggravated in UCP-2^-/-^ApoE^-/-^ mice.

**Fig 5:**
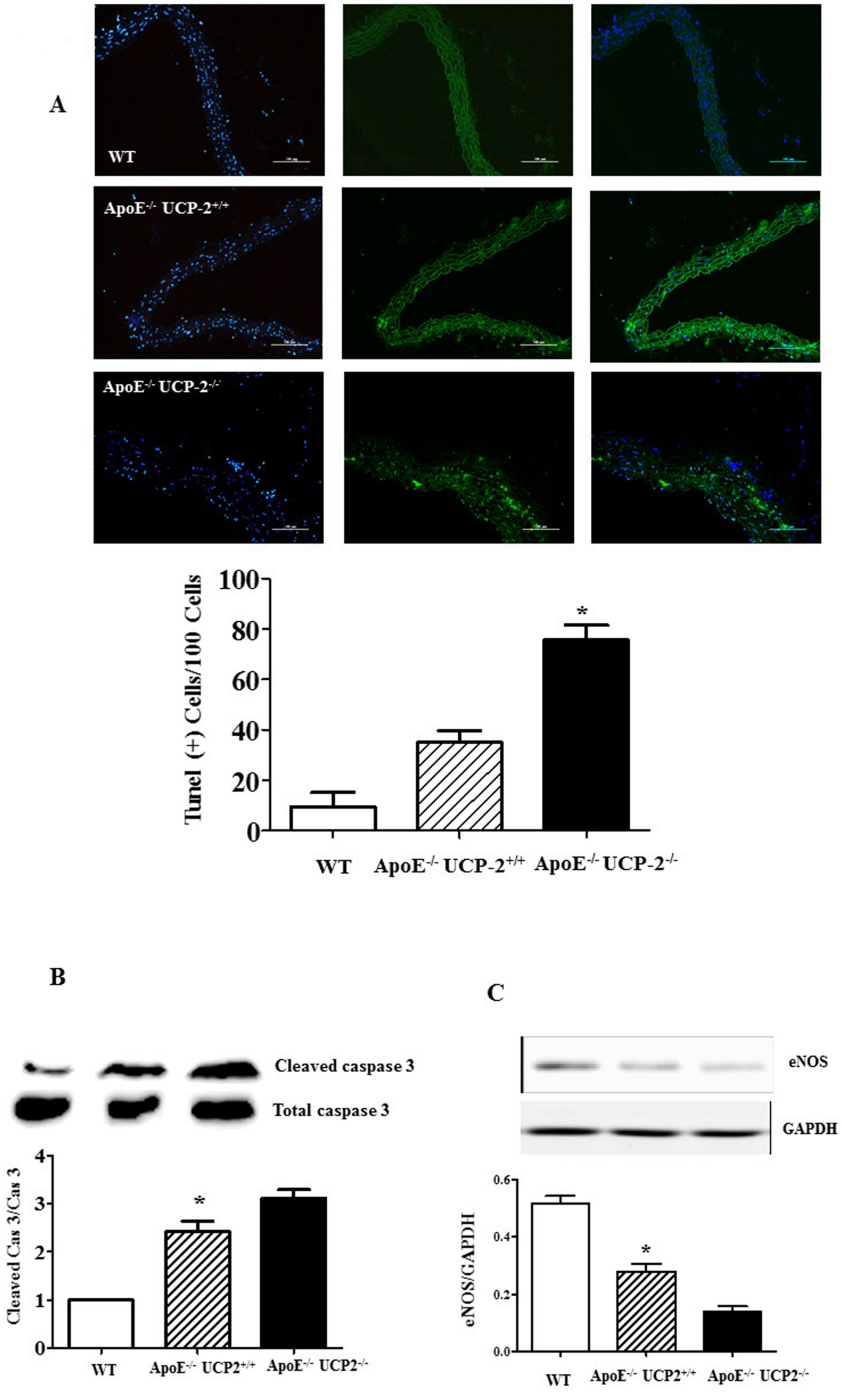
The effect of UCP-2- deficiency to VSMCs apoptosis. (**A**) Apoptotic VSMCs were determined by TUNEL staining. The percentage of TUNEL positive cells per 100 cells was calculated under a fluorescent microscope. These studies were repeated at least four times (*P<0.05 *vs*. others). (**B**) Caspase 3 activity was measured by immunoblotting analysis of cleaved caspase 3 and total caspase 3 expression. (*P<0.05 *vs*. others, n = 8). (**C**) eNOS protein expression was measured by immunoblotting analysis. Results are expressed as the ratio of eNOS and GAPDH. (*P<0.05 *vs*. others, n = 5).

Cells apoptosis could be an important negative factor in the pathological process of AAA [19,20]. Therefore, further studies checked the apoptosis by TUNEL assay, and the result found that Ang-Ⅱ induced apoptosis of VSMCs was increased in UCP-2^-/-^ApoE^-/-^ mice (**Fig 5A**). And the effect of UCP-2 in prevention of apoptotic VSMC death was further evident in the immunoblot of caspase 3, a key enzyme involved in execution of apoptosis. Clearly, the cleaved and active form caspase 3 was significantly increased in vascular tissue from UCP-2^-/-^ApoE^-/-^ mice (**Fig 5B**).

While UCP-2 has been found to increase endothelial nitric oxide synthase (eNOS) expression, inhibit apoptosis of cells from vessel walls and decrease ROS production [23], we measured the eNOS expression in aorta from different genotype mice and found it higher in WT mice than the UCP-2 KO mice before Ang-Ⅱ infusion (**S2 Fig**) and lower in UCP-2^-/-^ApoE^-/-^ mice than UCP-2^+/+^ApoE^-/-^ mice after 4 weeks Ang-Ⅱ treatment (**Fig 5C**), indicating that UCP-2 deficiency impaired the function of eNOS, and which could participates in formation of AAA.

## Discussion

UCP-2, as with the other members of UCPs, belong to the family of mitochondrial transporter proteins, is an important sensor and negative regulator of ROS production [24]. Therefore, UCP-2 could be a key molecular that resist the aneurysmal expansion. However, whether or not UCP-2 is involved in the disease progression of AAA is still unclear. The present study shows for the first time that deficiency of UCP-2 increased Ang-Ⅱ perfusion-induced aortic elastin degradation and destruction, and led to increased susceptibility of AAA. There is increasing evidence demonstrated the protective effect of UCP-2 on the vasculature against ROS-induced injuries. Kim *et al*. showed that up-regulation of UCP-2 could prevent the development of ROS-mediated atherosclerosis in patients with diabetes, obesity or hypertension[16]. And UCP-2 overexpression also ameliorates endothelial dysfunction with increased ROS [25].

Indeed, we also found the level of ROS was significantly increased in aortic tissue from UCP-2^-/-^ApoE^-/-^ mice, indicating the susceptibility of AAA is associated with the antioxidative effect of UCP-2. Furthermore, Ang-Ⅱ stimulates VSMCs abnormality through various pathways involving the production of ROS [26]. And Ang-Ⅱ induced ROS generation is primarily thought to be associated with membrane-bound NADH-/NADPH oxidase activating [27,28]. Our data showed the NADPH oxidase activity and level of MDA was significantly higher in UCP-2^-/-^ApoE^-/-^ mice than UCP-2^+/+^ApoE^-/-^ or WT mice. Previous studies also found the UCP-2 inhibitor, Genipin, elevated the NADPH oxidase activity via increasing both NOX2 mRNA level and endoplasmic reticulum (ER) stress, showing the key role of UCP-2 in the control of NADPH oxidase, as ROS sources [29]. And the MDA concentration significantly increased after inhibiting UCP-2 by Genipin [30].

On the contrary, our data demonstrated that deficiency of UCP-2 decreased the increasing SOD activity in Ang-Ⅱ treated ApoE^-/-^ mice although it still remains inconsistent about the interact between UCP-2 and SOD. A research from Ge *et al*. reported the SOD activity has an increasing tendency after UCP-2 inhibiting by Genipin in a dose-dependent manner [30]. And UCP-2, as mitochondrial anion transporter, could be one critical mediator in the control of mitochondrial ROS production and uncouple ATP synthesis from the oxidative phosphorylation pathway, which is regulated by PPARα and PGC-1α activation[31–33]. The mechanism of UCP-2 regulating SOD activity is still unclear, and need further study.

Besides, vascular smooth muscle cells apoptosis leads to aortic aneurysms and dissections. [19,20]. And our studies found that deficiency of UCP-2 increased apoptosis within the aortic wall. Wall stress due to the vessel’s expanding geometry significantly contributes to eventual rupture of AAA[34]. VSMC apoptosis decreased the aortic wall strength. AAA growth is accompanied by increasing wall stress and decreasing aortic strength. And enhanced wall stress may still occur due to early aortic biomechanical alterations i.e., VSMC apoptosis [35,36]. And previous studies show that UCP-2 overexpression in the aorta attenuate vascular damage via NO-dependent pathway[15]. UCP-2 regulates apoptosis in different cell lines. In our research, UCP-2 might prevent VSMCs apoptosis in AAA. And UCP-2 may protect cardiomyocytes by downregulating programmed cell death[37], whereas UCP-2-deficiency suppresses apoptosis in pulmonary artery smooth muscle cells (PASMCs)[38]. The PASMCs mitochondrial are different than the systemic vascular smooth muscle cell mitochondrial, which exists in a much more oxidized environment [39]. UCP-2 might play different roles in VSMCs apoptosis from different tissue.

Moreover, our studies indicated deficient of UCP-2 induced a significant decrease in eNOS protein expression, compared with WT of ApoE knockout mice. Nitric oxide (NO) produced by eNOS is one of the physiologically most important regulators of vascular function. Increasing superoxide production and decreasing NO production via eNOS uncoupling mediates AAA formation [22]. Previous *in vivo* studies using ApoE/eNOS dual deficient mice also demonstrated AAA development [40,41]. And activation of eNOS might reduce aortic inflammation and apoptosis, via NO pathway [42]. Thus, the anti-oxidants and anti-apoptosis effect of UCP-2 could be associated with eNOS.

In conclusion, our studies showed that UCP-2 protein and mRNA expression were significantly higher in Ang-Ⅱ-induced AAA of mice, and deficiency of UCP-2 increased susceptivity and severity of AAA via elevated ROS level and VSMCs apoptosis, indicating UCP-2 could been an anti-oxidants and anti-apoptosis factor in preventing AAA.

## Supporting information

S1 Fig The time course of systolic blood pressure. The mice were anesthetized with pentobarbital and BPs measured from the tail artery. BPs were obtained after a 1 hour stabilization period (n = 8).
(TIF)

S2 Fig The basal protein expression of eNOS in aorta from WT or UCP-2 knockout mice by immunoblotting. Results are expressed as the ratio of eNOS and GAPDH. (n = 5, *P<0.05 vs. others).
(TIF)
