# Supplementary figures and images for: UCP-2 is involved in angiotensin-II-induced abdominal aortic aneurysm in apolipoprotein E-knockout mice
Source: PLoS One. 2017 Jul 6;12(7):e0179743. doi: 10.1371/journal.pone.0179743 (PMC5500278; doi:10.1371/journal.pone.0179743)

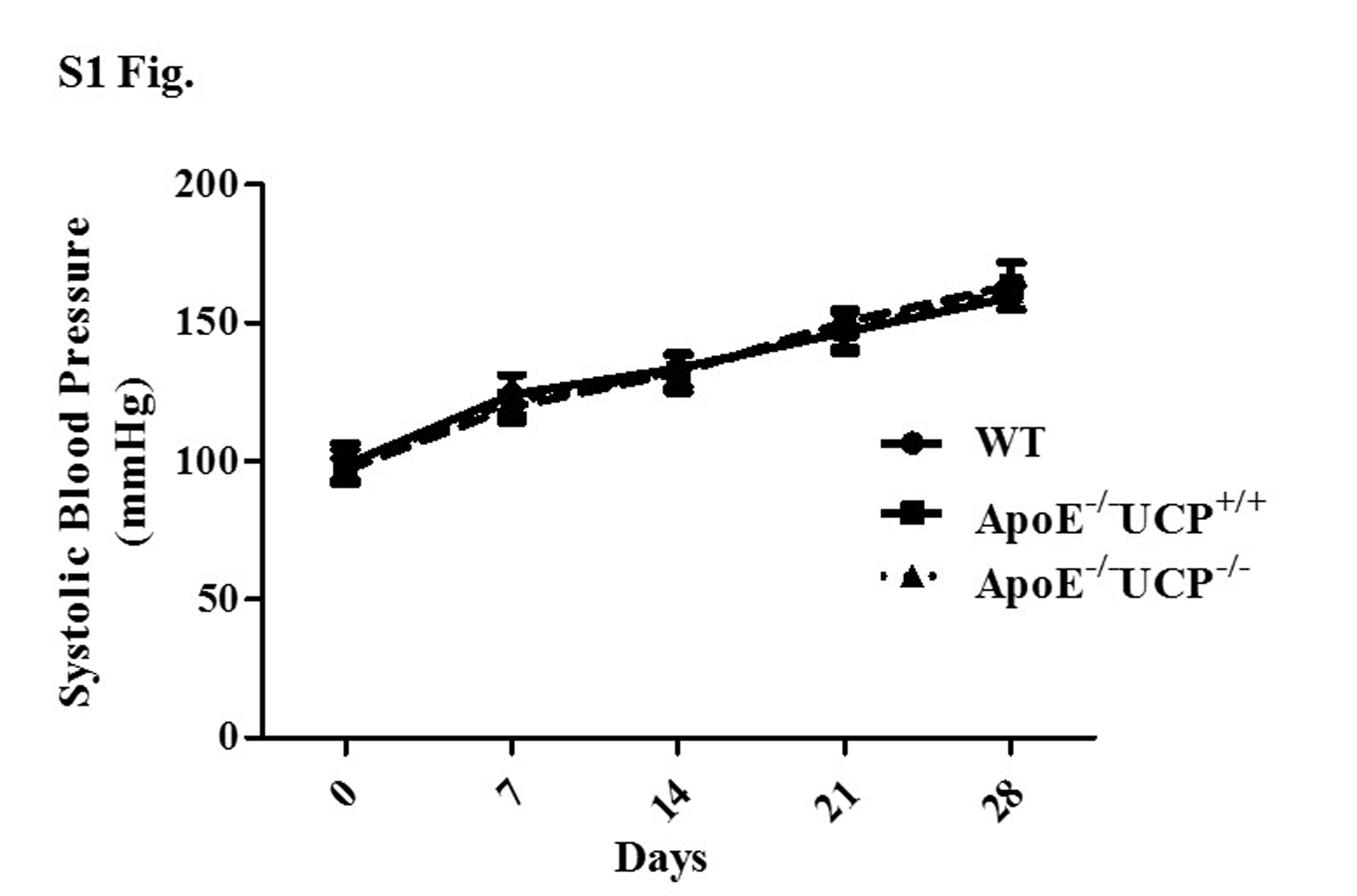

Supplement: S1 Fig — The mice were anesthetized with pentobarbital and BPs measured from the tail artery. BPs were obtained after a 1 hour stabilization period (n = 8). (TIF) [file pone.0179743.s001.tif]

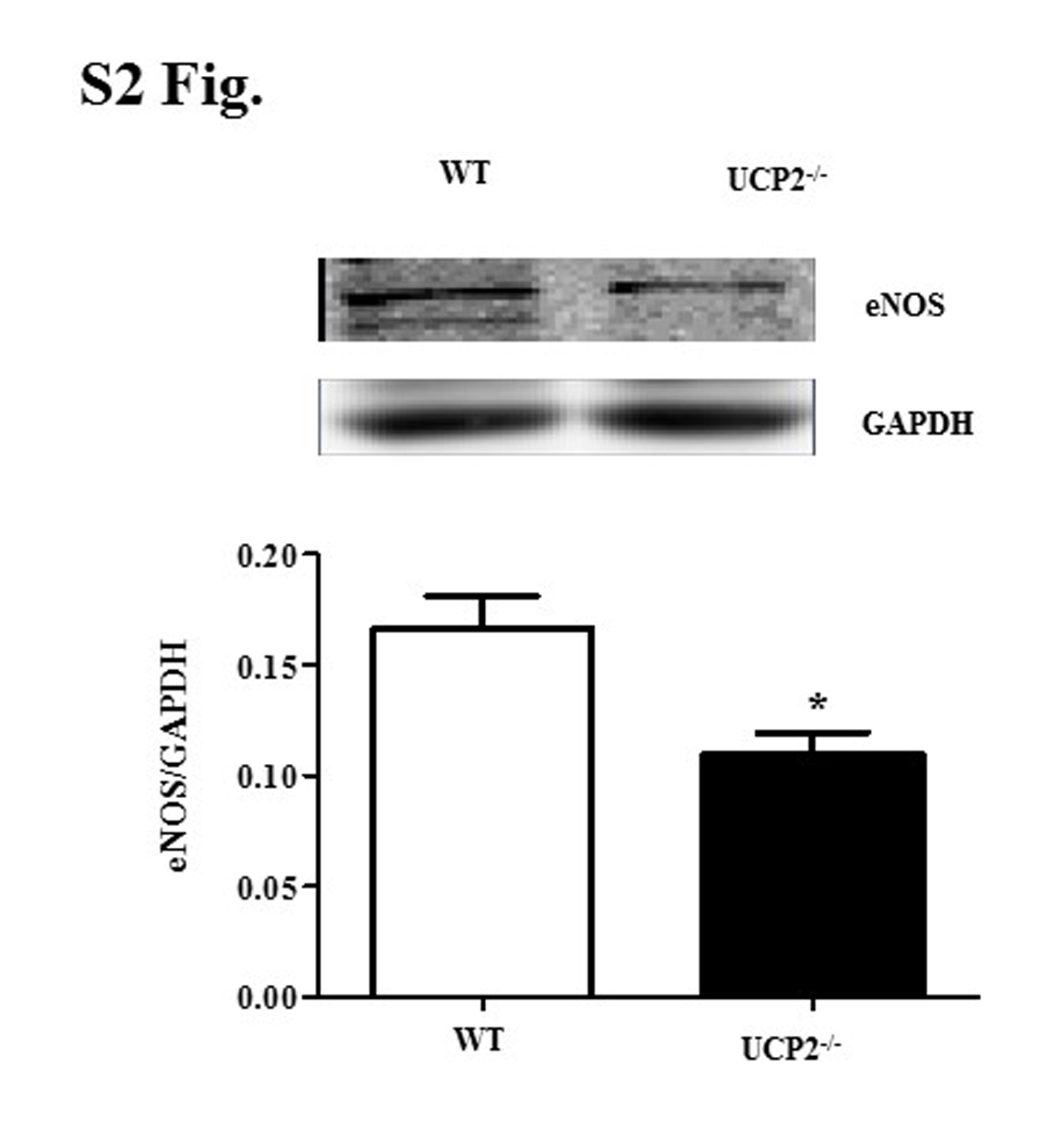

Supplement: S2 Fig — Results are expressed as the ratio of eNOS and GAPDH. (n = 5, *P<0.05 vs. others). (TIF) [file pone.0179743.s002.tif]
